# Cholesterol-Conjugated Polyion Complex Nanoparticles for Combination Delivery of Hydrophobic Paclitaxel and Hydrophilic miR-34a for Colon Cancer Therapy

**DOI:** 10.3390/ijms26167965

**Published:** 2025-08-18

**Authors:** Arjaree Jobdeedamrong, Hye Jin Yoo, Hosun Jung, Chiravoot Pechyen, Sitakan Natphopsuk, Peerapat Thongnuek, Seok Jeong, Junghan Lee, Su-Geun Yang

**Affiliations:** 1 BK21 FOUR Program in Biomedical Science and Engineering, Department of Biomedical Science, Inha University College of Medicine, Incheon 22332, Republic of Korea; arjaree.j@inha.ac.kr (A.J.); hyejin_yoo@inha.ac.kr (H.J.Y.); wjdghtjs1234@naver.com (H.J.); 2 Translational Research Center, Biomedical Research Institute, Inha University Hospital, Inha University College of Medicine, Incheon 22332, Republic of Korea; inos@inha.ac.kr; 3 Thammasat University Center of Excellence in Modern Technology and Advanced Manufacturing for Medical innovation, Thammasat University, Pathumthani 12120, Thailand; cpechyen@tu.ac.th (C.P.); sitakan@tu.ac.th (S.N.); 4 Department of Materials and Textile Technology, Faculty of Science and Technology, Thammasat University, Pathumthani 12120, Thailand; 5 Chulabhorn International College of Medicine, Thammasat University, Pathumthani 12120, Thailand; 6 Biomedical Materials and Devices for Revolutionary Integrative Systems Engineering Research Unit (BMD-RISE), Faculty of Engineering, Chulalongkorn University, Bangkok 10330, Thailand; peerapat.t@chula.ac.th; 7 Division of Gastroenterology, Department of Internal Medicine, Inha University Hospital, Inha University College of Medicine, 27 Inhang-ro, Jung-gu, Incheon 22332, Republic of Korea; 8 Department of Biomedical Science, Inha University College of Medicine, 366 Seohae-daero, Jung-gu, Incheon 22332, Republic of Korea

**Keywords:** cationic polymer systems, colorectal cancer model, dual-drug delivery, gene–chemo combination, gene transfection efficiency, miR-34a co-delivery, paclitaxel encapsulation, polyethylenimine nanocomplex

## Abstract

In recent years, combination chemotherapy with therapeutic nucleic acids has emerged as a promising strategy to enhance the effectiveness of cancer therapy. However, developing an effective co-delivery system to simultaneously transport both chemotherapeutic drugs and nucleic acids remains challenging. Herein, we fabricated cholesterol-conjugated polyion complex nanoparticles (PCNs) for combination delivery of hydrophobic paclitaxel (PTX) and hydrophilic miR-34a. Cholesterol was conjugated to polyethylenimine (PEI) and hyaluronic acid (HA), producing C–PEI and C–HA, respectively. PTX was initially encapsulated within the hydrophobic core formed by the self-assembly of C–HA and C–PEI, yielding polyion complex nanoparticles (PTX@C–HA/C–PEI PCNs). Subsequently, the negatively charged miR-34a was electrostatically complexed with the cationic C–PEI moieties to generate miR-34a/PTX@C–HA/C–PEI PCNs. These PCNs exhibited a nanoscale structure with a uniform size distribution and demonstrated low cytotoxicity in colon cancer cells. Fluorescence microscopy confirmed efficient cytosolic delivery of C–HA/C–PEI PCNs in colon carcinoma cells. Furthermore, combination delivery of PTX and miR-34a using C–HA/C–PEI PCNs exhibited significantly enhanced transfection efficiency and cellular uptake for human colon cancer cells. Notably, PTX/miR-34a@C–HA/C–PEI PCNs effectively downregulated critical oncogenic targets, including *Notch1*, *Snail1*, and *BCL-2*, resulting in reduced cancer cell migration and proliferation. These findings indicate that PTX/miR-34a@C–HA/C–PEI PCNs hold significant potential as an innovative combination delivery platform, offering improved therapeutic efficacy for colon cancer therapy.

## 1. Introduction

Colorectal cancer (CRC) is among the leading causes of cancer-related deaths worldwide, with a rising incidence particularly observed in developing countries [1,2,3]. This upward trend has been largely attributed to lifestyle changes, particularly the adoption of Western dietary patterns [4]. Such diets contribute to gut microbiota imbalances, which play a crucial role in the carcinogenesis of CRC [5,6]. Chemotherapy is commonly employed as a first-line treatment prior to surgical intervention, primarily because of its cost-effectiveness and proven therapeutic efficacy [7,8].

Paclitaxel (PTX), a conventional chemotherapeutic agent commonly selected for colon cancer treatment, exerts its anticancer effects by stabilizing microtubules during mitosis, thereby inducing apoptosis in cancer cells [9]. However, the clinical application of PTX is hindered by its inherent hydrophobicity and severe systemic toxicity, which limit its therapeutic index [10,11,12]. To address these limitations, nanodrug delivery systems have emerged as a promising strategy to enhance PTX’s solubility, improve targeted delivery, and minimize off-target effects [13].

In parallel, microRNA (miRNA) is a class of noncoding RNA that controls mRNA’s stability and translation. It plays pivotal roles in various physiological and pathological processes, including cellular proliferation, differentiation, and apoptosis, and is frequently dysregulated in cancer [14,15,16]. Among the types of microRNA, miR-34a is particularly notable for its critical role in the genesis and progression of human cancer cells, where its expression is frequently downregulated [17,18]. Restoration of miR-34a expression has been shown to induce apoptosis and suppress tumor progression, making it an attractive candidate for miRNA-based anticancer therapies [19]. Notably, miR-34a directly targets CD44, a cell surface glycoprotein involved in tumorigenesis and metastasis, making it a compelling candidate for cancer therapy [20,21]. However, the therapeutic application of miR-34a remains challenging because of its inherent instability and poor membrane permeability, both of which significantly limit its efficiency in tumor cells. To enhance the delivery efficiency of miR-34a, various nanocarrier systems, including viral vectors, micelles, and liposomes, have been explored [22]. Despite these advancements, the use of miR-34a as a standalone therapeutic remains inadequate, as cancer cells can activate compensatory pathways that allow them to evade miR-34a-induced apoptosis. Therefore, a combinatorial therapeutic strategy that integrates miR-34a with conventional chemotherapeutic agents, such as PTX, is considered essential to achieve a more robust and sustained antitumor response [23].

**Scheme 1:**
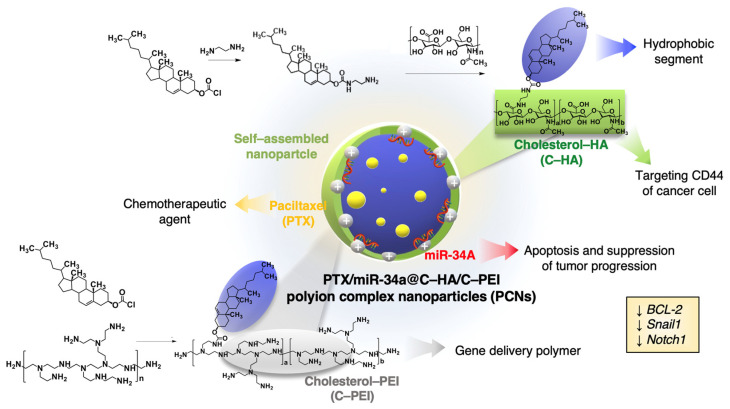
Schematic illustration of the polyion complex nanoparticles (PCNs) system. Cholesterol-modified hyaluronic acid (C–HA) and cholesterol-modified polyethyleneimine (C–PEI) self-assemble into a nanoscale delivery platform (PTX/miR-34a@C–HA/C–PEI PCNs) encapsulating paclitaxel (PTX) as a chemotherapeutic agent and miR-34a as a gene therapeutic. C–HA targets CD44 receptors on cancer cells, enhancing the delivery specificity, while miR-34a downregulates oncogenic markers (*BCL-2*, *Snail1*, *Notch1*), promoting apoptosis and suppressing tumor progression. Arrows indicate the direction of gene expression downregulation.

In this study, we prepared novel polyion complex nanoparticles (PCNs) composed of polycationic polyethylenimine (PEI) and polyanionic hyaluronic acid (HA) after conjugation of each polymer with cholesterol (C–PEI and C–HA) (Scheme 1). PEI, a cationic polymer, is widely recognized for its capability for nucleic acid delivery due to its strong electrostatic interactions with negatively charged nucleic acids and its capacity to facilitate endosomal escape [24,25]. In this system, PEI enabled the efficient complexation of miR-34a, a tumor-suppressive microRNA with demonstrated therapeutic efficacy in colorectal cancer. HA, a negatively charged linear polysaccharide, was integrated into the formulation to enhance colloidal stability of PCNs and provide active tumor-targeting capabilities through specific binding to CD44, a receptor overexpressed in many cancer types, including colon cancer [26,27]. To facilitate the co-delivery of hydrophobic PTX and hydrophilic miR-34a, both PEI and HA were chemically modified with hydrophobic cholesterol, thereby promoting their self-assembly into stable, core–shell-structured nanoparticles and improving the encapsulation efficiency of both therapeutic agents [28].

The physicochemical properties of the nanoparticles were characterized in terms of the particle size, morphology, surface charge, encapsulation efficiency, and drug loading content. The successful incorporation of miR-34a into the nanoparticles was confirmed by gel electrophoresis. In vitro transfection studies were conducted to evaluate the cellular uptake and delivery efficiency of miR-34a in murine colon carcinoma cells. To assess the therapeutic potential of the co-delivery system, we performed wound healing assays to evaluate cell migration and colony-forming assays to assess its long-term proliferative capacity. Additionally, gene silencing activity of the co-delivered miR-34a was assessed by gene expression analysis. These experiments aimed to investigate the biological impact of PTX and miR-34a co-delivered by the cholesterol-hyaluronic acid/cholesterol-polyethylenimine polyion complex nanoparticles (C–HA/C–PEI PCNs) for chemotherapeutic and gene therapy applications in colorectal cancer.

## 2. Results and Discussion

### 2.1. Preparation of Polyion Complex Nanocarriers

To enhance the therapeutic effects of the hydrophobic PTX and hydrophilic miR-34a in combination cancer treatment, it is crucial to design a co-delivery system using nanomaterials that improves the circulation time, promotes localized drug distribution, and minimizes off-target effects in non-tumor tissues. In this study, PEI was selected as a cationic polymer for its ease of surface functionalization and its ability to form stable ionic complexes with anionic therapeutic genes [29]. Additionally, HA, a negatively charged polysaccharide, was chosen for its excellent biocompatibility and selective targeting of CD44 receptors on cancer cells.

**Figure 1:**
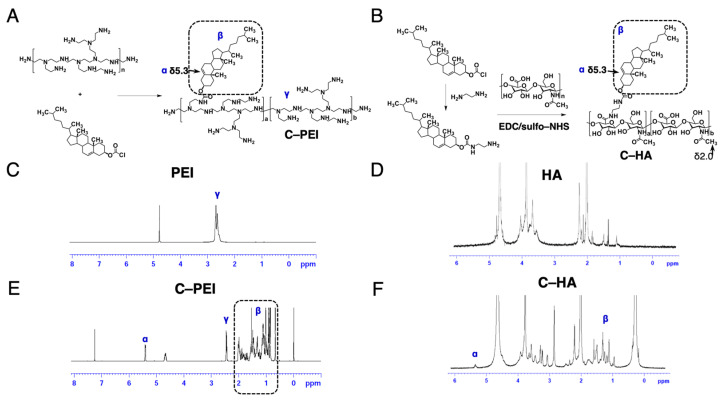
Chemical synthesis of cholesterol-conjugated polymers. (**A**) Schematic representation of the synthesis of cholesterol-modified polyethyleneimine (C–PEI) via nucleophilic substitution of linear PEI and cholesteryl chloroformate. (**B**) Schematic illustration of the synthesis of cholesterol-modified hyaluronic acid (C–HA) through amide bond formation between cholesteryl amine and HA using EDC/sulfo–NHS coupling chemistry. (**C**–**F**) ^1^H NMR spectra in D_2_O of (**C**) unmodified PEI, (**D**) HA, (**E**) C–PEI, and (**F**) C–HA. Characteristic proton signals corresponding to PEI (γ), cholesterol (α, β), and HA backbones are indicated.

To introduce a hydrophobic segment, the polymers were individually functionalized with cholesterol to form a hydrophobic structure that mimicked detergent behavior [30]. This modification was used to assemble a nanoscale drug delivery complex. C–PEI was synthesized via a condensation reaction between the amine groups of PEI and the acyl chloride groups of cholesteryl chloroformate (Figure 1A). The chemical structure of C–PEI was verified by ^1^H NMR spectroscopy (Figure 1C,E). In the ^1^H NMR spectra, signals corresponding to the protons in the β and α positions in cholesterol were observed at approximately 0.7–1.2 ppm and 5.3 ppm, respectively. Additionally, broad signals in the 2.0–2.5 ppm range, attributed to the γ protons of the PEI backbone, indicated successful cholesterol modification on the PEI main chain.

To synthesize C–HA, cholesteryl chloroformate was initially reacted with ethylenediamine to produce amine–Chol, which was subsequently conjugated to HA via EDC-mediated coupling (Figure 1B). The NMR spectrum (Figure S1) exhibited signals at 2.8 and 3.2 ppm, corresponding to the alkyl chain protons of amine–Chol. Moreover, signals at 5.32 ppm and 2.10 ppm, corresponding to the methine groups of cholesterol and the N-acetyl groups of HA, respectively, indicated the successful synthesis of C–HA (Figure 1D,F).

### 2.2. Characterization of Nanocarriers and Encapsulation Function for PTX and miR-34A

**Figure 2:**
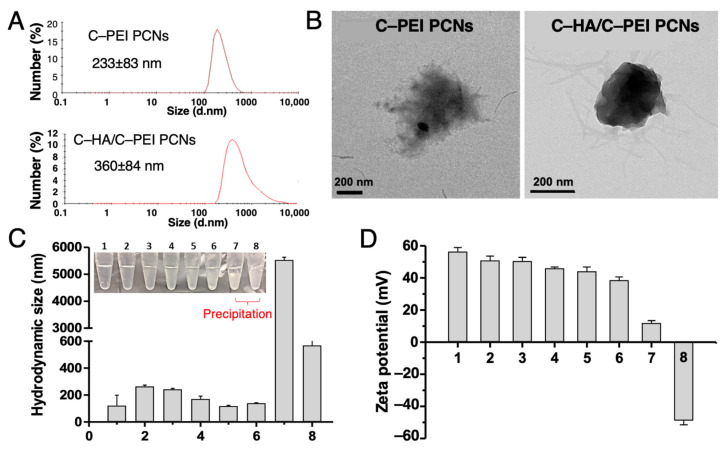
Characterization of C–PEI, C–HA, and C–HA/C–PEI PCNs. (**A**) Hydrodynamic diameter distribution of C–PEI (**top**) and C–HA (**bottom**) PCNs, as measured by DLS. (**B**) TEM images of C–PEI (**left**) and C–HA/C–PEI (**right**) PCNs. (**C**) Hydrodynamic diameter and (**D**) zeta potential of C–HA/C–PEI complex at different weight ratios of C–PEI to C–HA: 1:0 (1), 1:0.1 (2), 1:0.2 (3), 1:0.3 (4), 1:0.5 (5), 1:1 (6), 1:2 (7), and 0:2 (8).

The self-assembly behavior of C–HA and C–PEI in aqueous media was first examined. C–PEI and C–HA formed discrete nanoparticles in an aqueous solution at 5 mg/mL, with hydrodynamic diameters (*D*_h_) of 234 ± 83 nm and 360 ± 84 nm, respectively, as determined by dynamic light scattering (DLS) (Figure 2A). These results indicate that both components possess inherent self-assembling properties in aqueous solution. Transmission electron microscopy (TEM) revealed that C–PEI nanoparticles exhibited an irregular, low-density morphology (Figure 2B, left), whereas the C–HA/C–PEI polycationic nanocomplexes (PCNs) showed more condensed structures with rougher surface features (Figure 2B, right), consistent with the formation of polyelectrolyte nanocomplexes, due to the strong electrostatic interactions between C–PEI and C–HA.

To optimize the stoichiometry for complex formation, C–HA/C–PEI PCNs were formulated at varying C–PEI:C–HA weight ratios from 1:0 to 0:2. At ratios from 1:0 to 1:0.5, the *D*_h_ values remained between 150 and 250 nm (Figure 2C), and the dispersions remained visually turbid (inset, Figure 2C). While further increases in the HA content resulted in dramatic increases in the particle size, exceeding 5 μm at the 1:2 ratio and with visible precipitation, indicating aggregation and destabilization at a high HA content.

In addition, surface charge analysis employing the zeta potential (Figure 2D) demonstrated that formulations with C–PEI:C–HA ratios from 1:0 to 1:0.5 retained strongly positive surface potentials (+40 to +60 mV), consistent with colloidal stability. Ratios of 1:1 and 1:2 resulted in a marked reduction in the zeta potential (<+10 mV), likely due to charge neutralization by excess HA. Based on these findings, the 1:0.5 formulation was selected for subsequent encapsulation studies due to an optimal balance between the particle size, surface charge, and physical stability.

### 2.3. Optimization and Characterization of DNA and Paclitaxel Encapsulation in C–HA/C–PEI Polyion Complex Nanoparticles

DNA encapsulation within the C–PEI PCNs occurred through electrostatic interactions between the positively charged amine groups (N) of C–PEI and the negatively charged phosphate groups of DNA. PEI, a cationic polymer, interacts with negatively charged DNA or miRNA, forming stable nanoparticles that enhance the efficiency of nucleic acid delivery to cells. The nanoparticle structure protects the DNA from degradation by DNase, thereby contributing to high transfection efficiency. A general protocol for the complexation of nucleic acids with PEI is widely used in gene delivery applications [31].

**Figure 3:**
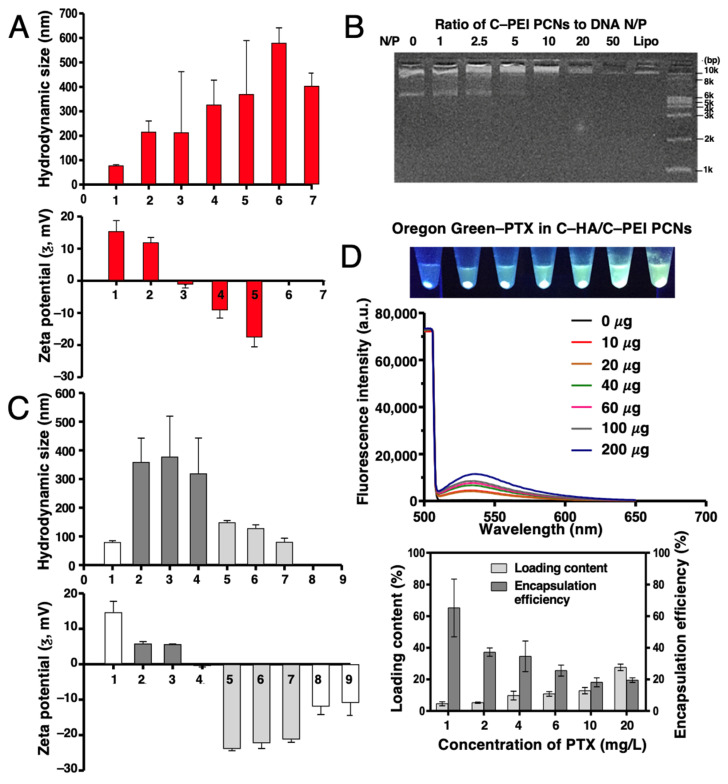
Characterization of nanoparticles for targeted gene delivery. (**A**) Hydrodynamic diameter (**top**) and zeta potential (**bottom**) of DNA@C–PEI PCNs at different C–PEI:DNA weight ratios: 1:0 (1), 1:0.25 (2), 1:0.5 (3), 1:0.75 (4), 1:1.24 (5), 1:2.48 (6), and 0:1.24 (7). (**B**) Agarose gel electrophoresis of DNA@C–PEI PCNs at increasing N/P ratios: DNA control (1); N/P = 1 (2), 2.5 (3), 5 (4), 10 (5), 20 (6), and 50 (7); lipofectamine/DNA ratio used as per manufacturer’s protocol (8); and DNA ladder (9). (**C**) Hydrodynamic diameter (**top**) and zeta potential (**bottom**) of C–HA/C–PEI PCNs at different C–HA:C–PEI:DNA weight ratios: 0.5:0:0 (1), 0.5:0.05:0.124 (2), 0.5:0.1:0.124 (3), 0.5:0.15:0.124 (4), 0:0.05:0.124 (5), 0:0.1:0.124 (6), 0:0.15:0.124 (7), 0:0:0.124 (8), and 0:0.15:0 (9). (**D**) Fluorescence spectra of Oregon Green-labeled paclitaxel (PTX) in centrifuged samples at varying PTX concentrations (0–200 µg/mL) (**top**) and corresponding loading content (%) and encapsulation efficiency (%) at PTX input concentrations of 1–20 mg/L (**bottom**).

To optimize DNA@C–PEI PCNs formation, various weight ratios of C–PEI to DNA were tested: 1:0, 1:0.25, 1:0.5, 1:0.7, 1:1.25, 1:1.5, and 0:1. As the DNA content increased, the *D*_h_ of the complex also increased (Figure 3A), whereas the zeta potential gradually decreased and eventually reversed from positive to negative values due to the negative charge of DNA. The stability of the DNA@C–PEI PCNs was further assessed by agarose gel electrophoresis (Figure 3B). At lower N/P ratios (1:1, 2.5:1, and 5:1), distinct DNA bands were visible, indicating partial complexation and free DNA migration. In contrast, at N/P ratios of 10:1, 20:1, and 50:1, no DNA bands were observed, confirming complete complexation that prevented DNA migration in the gel. In contrast, at lower N/P ratios, insufficient PEI resulted in incomplete complexation, allowing unbound DNA to leak from the complexes and appear as visible bands. Lipofectamine 2000 (Thermo Fisher, Waltham, MA, USA) was used as a positive control. Consequently, a C–PEI/DNA complex with an N/P ratio of 10:1 was selected for downstream studies as it provided complete DNA encapsulation. However, cationic C–PEI is prone to aggregation in biological environments due to nonspecific interactions with serum proteins, which can promote phagocytic clearance and complex disintegration, ultimately resulting in premature DNA release and degradation [32].

To address this limitation, HA, a naturally occurring component of the extracellular matrix, was introduced as a negatively charged surface coating to shield the cationic surface of the DNA@C–PEI PCNs. A HA coating was achieved via electrostatic assembly by varying the C–HA:C–PEI:DNA weight ratios. The *D*_h_ and zeta potential of the resulting complexes were measured to assess their stability and surface properties. Compared to uncoated DNA@C–PEI PCNs (1:10 N/P ratio), HA–coated formulations showed an increased *D*_h_ and a progressive decrease in the surface charge (Figure 3C and Table S1). When the weight ratio of C–HA to C–PEI/DNA reached 3:1, the zeta potential of the DNA@C–HA/C–PEI PCNs shifted from positive to negative (–1 ± 0.3 mV), signifying HA’s dominance on the surface of the PCNs. Consequently, a 3:1 weight ratio of C–HA to C–PEI/DNA was used for further experiments because of its nanoscale structure and lack of aggregation.

For the co-drug delivery, PTX was incorporated into the DNA@C–HA/C–PEI PCNs at an optimized ratio. PTX and DNA co-loaded into the C–HA/C–PEI PCNs were successfully formulated through self-assembly. 

To quantify the encapsulated PTX content within the nanocarrier, fluorescent dye-labeled PTX (Oregon Green 488-PTX, Thermo Fisher) was utilized. The fluorescence spectra of Oregon Green-labeled PTX in DMSO (Figure S2) exhibited concentration-dependent emission intensity, whereas the fluorescence signal in the aqueous supernatant after centrifugation of PCNs was negligible, indicating that the majority of PTX remained within the nanoparticle pellet. After purification, fluorescence spectra confirmed successful encapsulation across a range of PTX concentrations (Figure 3D, top). The loading content and encapsulation efficiency were quantified (Figure 3D, bottom). For example, with 20 mg/mL PTX, the encapsulation efficiency was 20% and the loading content was 30%, confirming the successful encapsulation of PTX in the DNA@C–HA/C–PEI PCNs.

### 2.4. In Vitro Cellular Uptake of Polyion Complex Nanoparticles

Despite extensive efforts to combat various forms of cancer, long-term tumor recurrence remains a major challenge. Recent studies have highlighted miR-34a as a potent tumor suppressor that directly targets CD44, thereby inhibiting the growth, differentiation, and metastasis of cancer stem cells [21]. These findings have sparked interest in developing miR-34a-based therapies as a promising approach for colorectal cancer treatment.

Although nucleic acid therapeutics involving the delivery of miR-34a via nanoparticles have been developed to suppress cancer cell growth, previous research indicates that such monotherapies pose a risk of tumor recurrence, as they primarily target cancer stem cells [33]. To achieve complete and sustained tumor eradication, it is imperative to employ more robust anticancer strategies that target all cancer cell populations.

In this study, we present a potent strategy to combat colorectal cancer through synergistic combination therapy that leverages the complementary effects of co-delivered agents. Our approach involves the simultaneous delivery of miR-34a and PTX using a co-delivery system designed to enhance the therapeutic efficacy. As an anti-oncogenic agent, miR-34a inhibits tumor growth by regulating key tumor-related genes, including Notch and CD44 [34,35,36]. Meanwhile, PTX, a mitotic inhibitor, disrupts the microtubule dynamics by stabilizing tubulin polymers, thereby interfering with cellular division processes [10,11].

**Figure 4:**
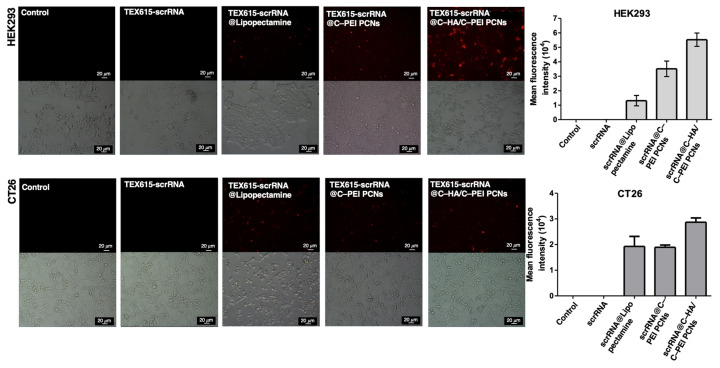
Cellular uptake of TEX615-scrambled RNA (scrRNA) delivered by various carriers in HEK293 and CT26 cells. Fluorescence microscopy (**top**) and corresponding bright-field (**bottom**) images of HEK293 (**top row**) and CT26 (**bottom row**) cells after 24 h incubation with TEX615-scrRNA formulations: free TEX615-scrRNA, TEX615-scrRNA/lipofectamine 3000, TEX615-scrRNA@C–PEI PCNs, and TEX615-scrRNA@C–HA/C–PEI PCNs. Red fluorescence indicates intracellular TEX615 signal. Mean fluorescence intensities were quantified using ImageJ (version 1.54g). Scale bars, 20 μm.

To ensure efficient delivery of these therapeutics, we engineered C–HA/C–PEI PCNs, which demonstrated high cellular uptake efficiency. The cellular uptake of the C–HA/C–PEI PCNs containing TEX615-scrambled RNA (scrRNA) (TEX615-scrRNA@C–HA/C–PEI PCNs) in HEK293 and CT26 cells was assessed using fluorescence microscopy. The resulting images revealed that the red fluorescent signals of the TEX615-RNA were predominantly localized within the cytoplasm (Figure 4), confirming the ability of the TEX615-scrRNA@C–HA/C–PEI PCNs to facilitate cytosolic delivery. Notably, the complex-treated group exhibited stronger fluorescence signals than the group treated with scrRNA alone, with the most pronounced signal observed in the C–HA/C–PEI PCNs-treated group. These results confirmed that these nanoparticles were effectively internalized by HEK293 and CT26 cells.

### 2.5. Evaluation of In Vitro Cytotoxicity and Gene Transfection Efficiency of Polyion Complex Nanoparticles

**Figure 5:**
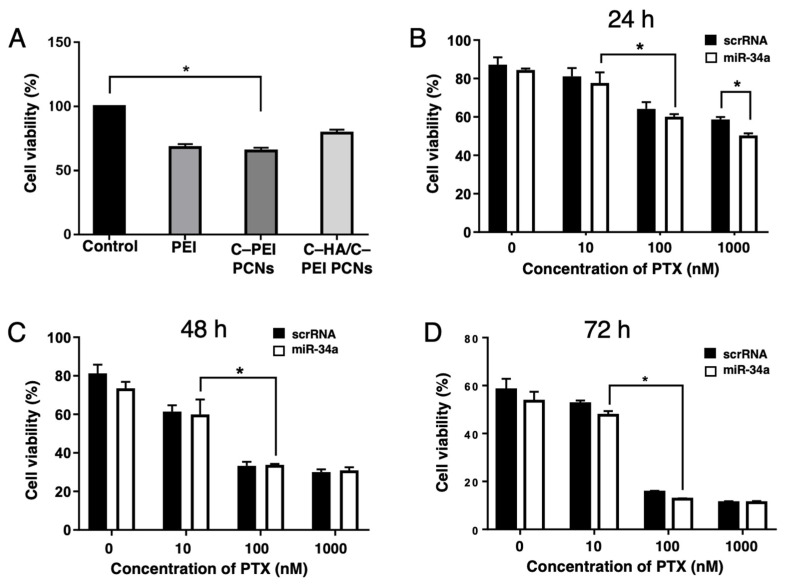
In vitro cytotoxicity of PTX and miR-34a co-delivery in HCT116 cells. (**A**) Cell viability of HCT116 cells treated with PEI, C–PEI PCNs, or C–HA/C–PEI PCNs without payload. (**B**–**D**) Cell viability following treatment with PTX encapsulated in miR-34a@C–HA/C–PEI PCNs, measured after 24 h (**B**), 48 h (**C**), and 72 h (**D**) incubation at 37 °C. Data presented as mean ± SD (n = 3). Statistical significance (*) indicated by *p* < 0.05 (Student’s *t*-test).

To further evaluate the cytotoxic effects of our co-delivery system comprising PTX and miR-34a in HCT116 cancer cells, we used an MTT assay. We first examined the biocompatibility by analyzing the viability of HEK293 cells following treatment with various formulations. The results indicated that coating these cells with C–HA/C–PEI PCNs significantly enhanced the viability of the HEK293 cells (Figure 5A), demonstrating that the HA layer effectively reduced the cytotoxicity of the complex. As depicted in Figure 5B, the cell viability in the PTX@C–HA/C–PEI-treated groups showed a slight dose-dependent decrease in both the scrambled RNA and miR-34a samples after 24 h. Notably, the viability of miR-34a-treated cells in the 1000 nM paclitaxel-treated group was significantly reduced compared to the untreated controls.

Moreover, with prolonged durations of exposure (48 and 72 h), a pronounced reduction in the cell viability was observed in both the scrambled RNA- and miR-34a-treated groups at lower PTX concentrations (100 nM) (Figure 5C,D). These findings indicate that combined treatment with miR-34a and PTX exhibits superior anticancer activity compared to miR-34a monotherapy (Figure S3). Furthermore, to evaluate the gene transfection and protein expression mediated by PCNs, GFP vector (pGFP)-loaded C–HA/C–PEI PCNs were used to treat HEY-T30 cells, and the GFP expression levels were measured by fluorescence microscopy. As shown in Figure S4, the GFP fluorescence intensity of the pGFP@C–HA/C–PEI PCNs-treated cells was similar to that of the positive control (pGFP/lipofectamine-treated cells), demonstrating its potential as a gene delivery carrier. This cooperative combination therapy is a promising strategy for enhancing the anticancer efficacy and minimizing the risk of tumor recurrence.

### 2.6. Cell Migration Assay of HCT116 Cells Treated with PTX and miR-34a Co-Delivery System

**Figure 6:**
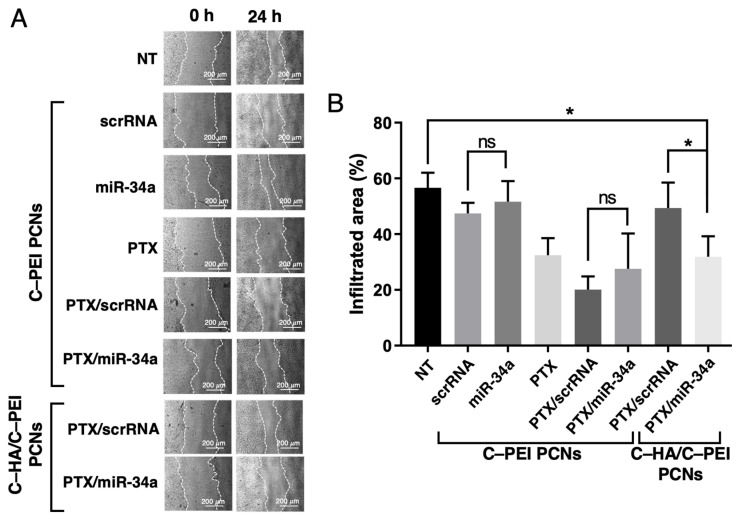
miR-34a’s inhibition of cell migration in HCT116 cells. (**A**) Representative micrographs from wound healing assays in HCT116 cells treated with nanoparticle formulations containing scrRNA, miR-34a, PTX, or combinations of them. Formulations were delivered using either C–PEI PCNs or C–HA/C–PEI PCNs. White dashed lines delineate wound margins at 0 h and 24 h. Scale bar = 200 μm. (**B**) Quantification of wound closure after 24 h, expressed as percentage of infiltrated area. Data are presented as mean ± SD (n = 3). Statistical comparisons were performed using Student’s *t*-test; *p* < 0.05 was considered significant (*), while ns = not significant.

The inhibitory effect of combined miR-34a and paclitaxel (PTX) treatment on cancer cell migration was assessed using a wound healing assay. HCT116 cells were transfected with miR-34a and treated with PTX encapsulated in C–HA/C–PEI or C–PEI PCNs. The wound widths were monitored at 0 and 24 h post-treatment, and representative images are shown in Figure 6A. The results demonstrated that PTX treatment significantly reduced the cell migration compared to that in the untreated controls after 24 h. Although the scratched area was reduced in the PTX/scrRNA@C–HA/C–PEI PCNs-treated group (Figure 6A, second row from the bottom), the PTX/miR-34a@C–HA/C–PEI PCNs-treated group (Figure 6A, bottom row) exhibited a relatively smaller reduction. The infiltrated area was quantified from the wound healing images (Figure 6B).

After 24 h, the cell migration rates were 56.6 ± 5.4% for the non-treated (NT) group, 47.4 ± 3.6% for the scrRNA@C–PEI PCNs group, and 51.7 ± 7.4% for the miR-34a@C–PEI PCNs group. These differences were not statistically significant, indicating that RNA delivery alone had a minimal impact on migration. In contrast, PCNs containing PTX markedly suppressed migration. Cells treated with PTX@C–PEI, PTX/scrRNA@C–PEI, and PTX/miR-34a@C–PEI exhibited reduced migration rates of 32.4 ± 6.2%, 20.1 ± 4.8%, and 27.5 ± 12.7%, respectively. These values were significantly lower than those of the NT and RNA-only groups (*p* < 0.05), confirming the anti-migratory effect of PTX.

When C–HA/C–PEI was used as the delivery vehicle, distinct effects were observed. The PTX/scrRNA@C–HA/C–PEI group showed a migration rate of 49.4 ± 9.2%, which was not significantly different from that of the NT group, suggesting that HA surface modification may have reduced the activity of PTX. However, the PTX/miR-34a@C–HA/C–PEI group exhibited a significantly lower migration rate of 31.9 ± 7.3%, indicating enhanced suppression of migration compared to that in both the NT and PTX/scrRNA@C–HA/C–PEI groups (*p* < 0.05). Overall, these results demonstrate that co-delivery of PTX and miR-34a via C–HA/C–PEI nanocarriers more effectively inhibits HCT116 cell migration than either agent alone or when the nanocarriers are delivered without HA modification.

### 2.7. Clonogenic Assay and Gene Expression Analysis

**Figure 7:**
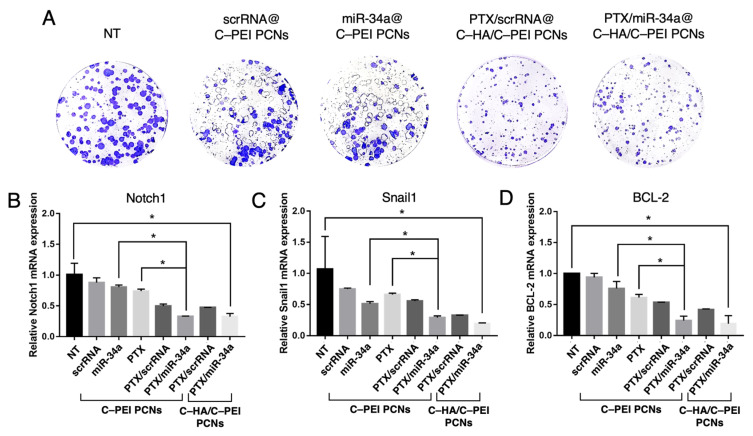
Clonogenic cell survival assay and RT-PCR analysis of gene expression in HCT116 cells treated with nanocarriers and combination treatment. (**A**) Representative images of crystal violet-stained colonies formed after 10-day incubation with different treatments: untreated (NT), scrRNA@C–PEI PCNs, miR-34a@C–PEI PCNs, PTX/scrRNA@C–HA/C–PEI PCNs, and PTX/miR-34a@C–HA/C–PEI PCNs. (**B**–**D**) Quantitative RT-PCR analysis of mRNA expression levels of (**B**) *Notch1*, (**C**) *Snail1*, and (**D**) *BCL-2* in treated HCT116 cells. Expression levels were normalized to GAPDH and are presented as relative to those of NT control group. All data are shown as mean ± SD (n = 3). Statistical analysis was performed using Student’s *t*-test; *p* < 0.05 indicates statistical significance (*).

To assess the effect of the co-delivery system on cell proliferation, a clonogenic assay was performed using crystal violet staining (Figure 7A). The assay revealed a substantial reduction in both the size and number of cell colonies after treatment with the dual-drug combination of miR-34a and PTX, suggesting that the co-delivery system effectively impaired cancer cell survival and proliferation.

To further validate the cellular effects of this co-delivery system, we examined the expression of the target genes *Notch1*, *Snail1*, and *BCL-2* in HCT116 cancer cells using a real-time reverse transcriptase polymerase chain reaction (RT-PCR). *Notch1*, *Snail1*, and *BCL-2* were selected due to their critical involvement in metastasis, CRC progression, and resistance to treatment. *Notch1* is essential for maintaining cancer stem cells and promoting tumorigenesis [37]. *Snail1* drives the epithelial–mesenchymal transition (EMT), facilitating tumor cell invasion and metastasis [38]. *BCL-2* prevents apoptosis, contributing to chemoresistance in CRC [39]. As shown in Figure 7B, the *Notch1* expression was downregulated by approximately 2– to 2.5–fold following treatment with the combination (PTX/miR-34a) compared to treatment with either drug alone (PTX or miR-34a). Moreover, the co-delivery of PTX and miR-34a using the C–HA/C–PEI PCNs further reduced the *Notch1* expression by nearly 3–fold relative to that of the control group.

Similarly, the expression of *Snail1* and *BCL-2* was significantly suppressed by the combination treatment, as illustrated in Figure 7C,D. The PTX/miR-34a@C–HA/C–PEI PCNs decreased the *Snail1* expression by 18% and the *BCL-2* expression by 19%. Collectively, these findings indicate that combination treatment exerted a more profound inhibitory effect on downstream target genes than single-drug treatments, highlighting the enhanced therapeutic potential of the proposed co-delivery system.

Compared to a previously reported dual-delivery system based on cationic solid lipid nanoparticles, which achieved high encapsulation efficiencies (~93% for PTX and ~95% for miR-34a) and strong antitumor activity in a melanoma model [23], our HA–Chol/PEI–Chol nanoparticles offer a biocompatible and innovative design for colorectal cancer. The HA coating promotes CD44-mediated uptake while attenuating the PEI-induced cytotoxicity. Despite a lower zeta potential, our system enabled effective RNA delivery and robust inhibition of cancer cell proliferation and migration, underscoring the significance of rational nanoparticle compositions and targeting in optimizing therapeutic performance.

In addition, the functional delivery outcomes are consistent with the proton sponge behavior of PEI, which is widely recognized to facilitate endosomal escape via buffering-induced osmotic swelling and vesicle ruptures. In particular, recent in situ studies using co-localized fluorescent pH sensor microcapsules confirmed that PEI can buffer lysosomal acidification within the endocytic vesicle, thereby enabling cytoplasmic release of encapsulated cargo [40]. In our system, the buffering ability of PEI, together with the membrane-interactive properties of cholesterol, likely contributes to the enhanced transfection and therapeutic activity observed.

## 3. Materials and Methods

### 3.1. Materials

Hyaluronic acid (HA, average molecular weight of 200 kDa, Lifecore Biomedical, Inc., Chaska, MN, USA), 1-Ethyl-3-(3-dimethylaminopropyl)carbodiimide (EDC, TCI, Tokyo, Japan, >98%), sulfo-N-hydroxysuccinimide (sulfo-NHS, Sigma-Aldrich, Saint Louis, MO, USA, >98%) tetrahydrofuran (THF, Daejung, Gyeonggi-do, Republic of Korea, >99%), polyethyleneimine (PEI, average molecular weight of 25 kDa, Sigma-Aldrich) cholesteryl chloroformate (Sigma-Aldrich, 95%), deoxyribonucleic acid sodium salt from Salmon testes (DNA, Sigma-Aldrich, 15.3 A260 unit/mg solid), miR-34a (BIONEER Corporation, Daejeon, Republic of Korea), and anhydrous methylene chloride (Daejung, 99.9%) were all used as received. Deionized water was used in all the experiments.

### 3.2. Synthesis of Amine-Modified Cholesterol

Cholesteryl chloroformate reacted with ethylenediamine to synthesize amine-modified cholesterol. Briefly, cholesteryl chloroformate (2.25 g, 5 mmol) was dissolved in anhydrous toluene (50 mL) and added dropwise to a solution of ethylenediamine (16.7 mL, 250 mmol) in 150 mL of toluene over 10 min under constant stirring at 0 °C. The reaction mixture was then stirred overnight at 25 °C.

Following the reaction, unreacted ethylenediamine was thoroughly removed by washing with distilled water, the mixture was dried over anhydrous magnesium sulfate, and the solvent evaporated under reduced pressure. The resulting residue was dissolved in 100 mL of a dichloromethane/methanol (1/1, *v*/*v*) solution. Byproducts were removed using a syringe filter (MWCO = 1 μm, PTFE, Whatman, NJ, USA). The solvent was removed by rotary evaporation and the structure of the resulting product was confirmed by ^1^H-NMR.

### 3.3. Synthesis of Cholesterol-Conjugated Hyaluronic Acid (C–HA)

Amine-functionalized cholesterol was conjugated to hyaluronic acid (HA) via EDC–mediated coupling between the amine groups of amine-modified cholesterol and the carboxyl groups of hyaluronic acid. Briefly, HA (100 mg, 244 μmol) was dissolved in 10 mL of distilled water, followed by the addition of EDC (93.5 mg, 0.602 mmol) and sulfo–NHS (106 mg, 0.488 mmol). The reaction mixture was stirred for 30 min to activate the carboxyl groups of HA. Subsequently, a solution of amine-modified cholesterol (48.8 μmol) in 10 mL of THF was added dropwise into the HA solution, and the mixture was stirred for 18 h at room temperature. Following the reaction, the product was purified by dialysis (MWCO = 12 kDa, Spectrum Laboratories, Inc., Rancho Dominguez, CA, USA) against water/THF (1:1, *v*/*v*) for 2 days, followed by dialysis against water for 1 day prior to lyophilization.

### 3.4. Synthesis of Cholesterol-Conjugated Polyethyleneimine (C–PEI)

Cholesteryl chloroformate (36 mg, 80 μmol) was added to a 150 mL solution of branched polyethylenimine (PEI, 1 g) in anhydrous methylene chloride, and the reaction mixture was stirred overnight at 25 °C. Subsequently, the organic solvent was removed by rotary evaporation. The resulting product was purified by dialysis (MWCO = 12 kDa) against water for 3 days before lyophilization.

### 3.5. Loading of Paclitaxel (PTX) and Nucleic Acids into C–PEI and C–HA Polyion Complex Nanoparticles (C–HA/C–PEI PCNs)

Paclitaxel (PTX) and nucleic acids (either DNA or miR-34a) were incorporated into the C–HA/C–PEI PCNs via self-assembly. First, PTX was dissolved in DMSO at a concentration of 20 mg/mL. A certain volume of the PTX solution was added to C–PEI and C–HA (at a 3:1 weight ratio of C–HA:C–PEI) dissolved in nuclease-free water, and the mixture was sonicated to form a hydrophobic PTX@C–HA/C–PEI complex. A general protocol for the complexation of nucleic acids with PEI is widely used in gene delivery applications [29]. PEI, a cationic polymer, interacts with negatively charged DNA or miRNA, forming stable nanoparticles that enhance the efficiency of nucleic acid delivery to cells. Therefore, miR-34a in nuclease-free water was then added dropwise to the PTX@C–HA/C–PEI complex at a weight ratio of 1:10 (miR-34a:C–PEI), followed by vortexing for 3 min to obtain the PTX/miR-34a@C–HA/C–PEI PCNs.

### 3.6. Characterization of C–HA/C–PEI PCNs

The chemical structures were confirmed using ^1^H NMR spectroscopy (Bruker Avance III 400 MHz, Bruker, Billerica, MA, USA). The hydrodynamic diameters and zeta potentials of the nanoparticles were measured by dynamic light scattering (DLS, Zetasizer Nano ZS, Malvern Instruments Ltd., Worcestershire, UK) at 25 °C. The morphology of the nanoparticles was examined by transmission electron microscopy (TEM, JEOL, JEM-2100F, Tokyo, Japan). For sample preparation, diluted nanoparticle dispersions were placed on copper grids for TEM measurements. The encapsulation efficiency (EE%) and loading content (LC%) of PTX in the PCNs were determined using fluorescence spectroscopy. After centrifugation to separate unencapsulated PTX, the amount of free PTX in the supernatant was quantified by fluorescence measurement. The EE% was calculated as the percentage of encapsulated PTX relative to the initial amount of PTX added, while the LC% was defined as the percentage of encapsulated PTX relative to the total weight of the nanoparticles.

### 3.7. Cell Culture

CT-26 and HCT-116 cells were cultured in Dulbecco’s Modified Eagle Medium (DMEM) supplemented with 10% fetal bovine serum (FBS) and 1% penicillin/streptomycin (P/S) and incubated at 37 °C under 5% CO_2_ within a humidified atmosphere. The cells were grown to a 70% confluence before being split or harvested for further experiments.

### 3.8. Cell Viability Test

HCT116 cells (2 × 10^4^ cells/well) were seeded in 96-well plates and incubated in DMEM containing 10% FBS. After attachment, the cells were treated with each formulation of the PCNs. The cell viability was assessed using a WST-1 assay kit (Takara Bio Inc., Shiga, Japan) at 24, 48, and 72 h post-treatment, following the manufacturer’s protocol.

### 3.9. Cellular Uptake

CT26 cells and HEK293 (4 × 10^4^ cells/well) were seeded onto 8-well chambered coverslips and allowed to adhere. They were subsequently treated with TEX615-labeled scrambled RNA (TEX615-scrRNA, 2 μg/mL), TEX615-scrRNA@C–PEI PCNs (2:8 weight ratio of TEX615-scrRNA:C–PEI), TEX615-scrRNA@C–HA PCNs (2:2.4 weight ratio of TEX615-scrRNA:C–HA), TEX615-scrRNA@C–HA/C–PEI PCNs (2:2.4:8 weight ratio of TEX615-scrRNA:C–HA:C–PEI), and TEX615-scrRNA/lipofectamine (2:12 weight ratio of TEX615-scrRNA:lipofectamine). After 24 h of incubation, the cells were fixed with 4% paraformaldehyde and stained with 4′,6-diamidino-2-phenylindole (DAPI). The stained samples were then examined under a confocal microscope (FV1000, Olympus, Tokyo, Japan).

### 3.10. Wound Healing Assay

HCT116 cells (1 × 10^5^ cells/well) were seeded into 24-well plates and transfected with various formulations, including PEI PCNs containing PTX (25 nM), scrambled RNA (scrRNA), miR-34a (100 nM), scrRNA/PTX, or miR-34a/PTX, as well as C–HA/C–PEI PCNs loaded with scrRNA/PTX or miR-34a/PTX. Both transfected and non-transfected cells were cultured in DMEM supplemented with 10% FBS and incubated at 37 °C with 5% CO_2_ for 24 h until reaching a 90–100% confluence. Linear scratches were created in the cell monolayer using a 100 μL pipette tip, after which the cells were maintained in serum-free DMEM. The wound healing process was observed under an optical microscope 24 h post-scratching. The extent of the wound closure was quantified by measuring the remaining wound area at each time point relative to the initial wound area to assess the cell migration and wound healing over time.

### 3.11. Colony-Forming Assay

HCT116 cells (2 × 10^3^ cells/well) were seeded into 6-well plates. The cells were subsequently transfected with PEI PCNs containing PTX (25 nM), scrRNA, miR-34a (100 nM), scrRNA/PTX, or miR-34a/PTX, as well as C–HA/C–PEI PCNs containing scrRNA/PTX or miR-34a/PTX. After transfection, the cells were incubated for 2 weeks in DMEM supplemented with 10% FBS and maintained at 37 °C with 5% CO_2_. After incubation, the colonies were washed twice with 1 mL of phosphate-buffered saline (PBS), fixed with 4% paraformaldehyde for 30 min at room temperature, and stained with 1 mL of crystal violet per well for 30 min at room temperature to visualize the colonies.

### 3.12. Reverse Transcription Polymerase Chain Reaction (RT-PCR) for Snail1, Notch1, and BCL-2

Total RNA from HCT116 cells was extracted using TRIzol^TM^ reagent following the manufacturer’s instructions. *Snail1*, *Notch1*, *BCL-2*, and *GAPDH* mRNA were amplified using a real-time one-step RT-PCR method and a One-Step SYBR RT-PCR kit (Meridian Bioscience, London, UK). The qPCR was performed according to the manufacturer’s protocol.

### 3.13. Statistical Analysis

All quantitative data in this study are presented as the mean ± the standard deviation (SD). Statistical analyses were performed using GraphPad Prism software (version 7). Differences between experimental groups were evaluated using Student’s *t*-test, and a *p*-value < 0.05 was considered statistically significant (*).

## 4. Conclusions

This study developed polyion complex nanocarriers (PCNs) composed of cholesterol-conjugated hyaluronic acid (C–HA) and cholesterol-conjugated polyethyleneimine (C–PEI) for the combinatorial delivery of paclitaxel (PTX) and miR-34a. These nanocarriers effectively reduced the drug-associated toxicity and enhanced the stability of the delivery system by co-encapsulating hydrophobic PTX and hydrophilic miR-34a within the C–HA/C–PEI PCNs. Co-loading was facilitated by hydrophobic core formation driven by cholesterol moieties and stabilized through electrostatic interactions. This design significantly improved the cellular uptake and transfection efficiency. As a result, co-delivery of PTX and miR-34a via cholesterol-conjugated PCNs enabled effective modulation of therapeutic gene expression in colorectal cancer cells, presenting a promising strategy for combinatorial cancer chemotherapy. Although the in vitro results are promising, in vivo studies would further strengthen the findings regarding its therapeutic efficacy, biodistribution, and pharmacokinetics. Future in vivo studies will be necessary to confirm its potential for clinical application.

## Data Availability

The original contributions presented in this study are included in the article/Supplementary Materials. Further inquiries can be directed to the corresponding author.

## Supplementary Materials

The following supporting information can be downloaded at https://www.mdpi.com/article/10.3390/ijms26167965/s1.

## Abbreviations

The following abbreviations are used in this manuscript:

C–HA	Cholesterol-conjugated hyaluronic acid
C–PEI	Cholesterol-conjugated polyethylenimine
C–HA/C–PEI PCNs	Cholesterol-conjugated polyethylenimine and cholesterol-conjugated hyaluronic acid polyion complex nanoparticles
CRC	Colorectal cancer
HA	Hyaluronic acid
PEI	Polyethylenimine
PCNs	Polyion complex nanoparticles
PTX	Paclitaxel

## References

[1] Arnold M., Sierra M.S., Laversanne M., Soerjomataram I., Jemal A., Bray F. (2017). Global patterns and trends in colorectal cancer incidence and mortality. Gut.

[2] Allemani C., Weir H.K., Carreira H., Harewood R., Spika D., Wang X.S., Bannon F., Ahn J.V., Johnson C.J., Bonaventure A. (2015). Global surveillance of cancer survival 1995–2009: Analysis of individual data for 25 676 887 patients from 279 population-based registries in 67 countries (CONCORD-2). Lancet.

[3] Argiles G., Tabernero J., Labianca R., Hochhauser D., Salazar R., Iveson T., Laurent-Puig P., Quirke P., Yoshino T., Taieb J. (2020). Localised colon cancer: ESMO Clinical Practice Guidelines for diagnosis, treatment and follow-up. Ann. Oncol..

[4] Kerr J., Anderson C., Lippman S.M. (2017). Physical activity, sedentary behaviour, diet, and cancer: An update and emerging new evidence. Lancet Oncol..

[5] Bullman S., Pedamallu C.S., Sicinska E., Claney T.E., Zhang X.Y., Cai D.N., Neuberg D., Huang K., Guevara F., Nelson T. (2017). Analysis of Fusobacterium persistence and antibiotic response in colorectal cancer. Science.

[6] Pleguezuelos-Manzano C., Puschhof J., Huber A.R., van Hoeck A., Wood H.M., Nomburg J., Gurjao C., Manders F., Dalmasso G., Stege P.B. (2020). Mutational signature in colorectal cancer caused by genotoxic pks(+) *E. coli*. Nature.

[7] Asare E.A., Evans D.B., Erickson B.A., Aburajab M., Tolat P., Tsai S. (2016). Neoadjuvant treatment sequencing adds value to the care of patients with operable pancreatic cancer. J. Surg. Oncol..

[8] Lee J.C., Ahn S., Paik K.H., Kim H.W., Kang J., Kim J., Hwang J.H. (2016). Clinical impact of neoadjuvant treatment in resectable pancreatic cancer: A systematic review and meta-analysis protocol. BMJ Open.

[9] Sati P., Sharma E., Dhyani P., Attri D.C., Rana R., Kiyekbayeva L., Büsselberg D., Samuel S.M., Sharifi-Rad J. (2024). Paclitaxel and its semi-synthetic derivatives: Comprehensive insights into chemical structure, mechanisms of action, and anticancer properties. Eur. J. Med. Res..

[10] Wall M.E., Wani M.C. (1996). Camptothecin and taxol: From discovery to clinic. J. Ethnopharmacol..

[11] Cavallaro G., Licciardi M., Caliceti P., Salmaso S., Giammona G. (2004). Synthesis, physico-chemical and biological characterization of a paclitaxel macromolecular prodrug. Eur. J. Pharm. Biopharm..

[12] Zhang B., Sun X.Y., Mei H., Wang Y., Liao Z.W., Chen J., Zhang Q.Z., Hu Y., Pang Z.Q., Jiang X.G. (2013). LDLR-mediated peptide-22-conjugated nanoparticles for dual-targeting therapy of brain glioma. Biomaterials.

[13] Sharifi-Rad J., Quispe C., Patra J.K., Singh Y.D., Panda M.K., Das G., Adetunji C.O., Michael O.S., Sytar O., Polito L. (2021). Paclitaxel: Application in modern oncology and nanomedicine-based cancer therapy. Oxid. Med. Cell. Longev..

[14] Dorton B.J., Elias K.M., Growdon W., Horowitz N.S. (2015). Biomarkers in Gynecologic Cancers Red cell distribution width (RDW) as a novel marker for predicting recurrence of high-grade cervical dysplasia or carcinoma in active smokers. Gynecol. Oncol..

[15] Kong Y.W., Ferland-McCollough D., Jackson T.J., Bushell M. (2012). microRNAs in cancer management. Lancet Oncol..

[16] Malumbres M. (2012). miRNAs versus oncogenes: The power of social networking. Mol. Syst. Biol..

[17] Zhang C.C., Mo R., Yin B.D., Zhou L.B., Liu Y.C., Fan J. (2014). Tumor suppressor microRNA-34a inhibits cell proliferation by targeting Notch1 in renal cell carcinoma. Oncol. Lett..

[18] Li X.J., Ren Z.J., Tang J.H. (2014). MicroRNA-34a: A potential therapeutic target in human cancer. Cell Death Dis..

[19] Bader A.G., Brown D., Winkler M. (2010). The Promise of MicroRNA Replacement Therapy. Cancer Res..

[20] Joshua B., Kaplan M.J., Doweck I., Pai R., Weissman I.L., Prince M.E., Ailles L.E. (2012). Frequency of cells expressing CD44, a Head and Neck cancer stem cell marker: Correlation with tumor aggressiveness. Head Neck.

[21] Liu C., Kelnar K., Liu B.G., Chen X., Calhoun-Davis T., Li H.W., Patrawala L., Yan H., Jeter C., Honorio S. (2011). The microRNA miR-34a inhibits prostate cancer stem cells and metastasis by directly repressing CD44. Nat. Med..

[22] Bu P., Chen K.-Y., Chen J.H., Wang L., Walters J., Shin Y.J., Goerger J.P., Sun J., Witherspoon M., Rakhilin N. (2013). A microRNA miR-34a-regulated bimodal switch targets Notch in colon cancer stem cells. Cell Stem Cell.

[23] Shi S., Han L., Deng L., Zhang Y., Shen H., Gong T., Zhang Z., Sun X. (2014). Dual drugs (microRNA-34a and paclitaxel)-loaded functional solid lipid nanoparticles for synergistic cancer cell suppression. J. Control. Release.

[24] Choosakoonkriang S., Lobo B.A., Koe G.S., Koe J.G., Middaugh C.R. (2003). Biophysical characterization of PEI/DNA complexes. J. Pharm. Sci..

[25] He Y.Y., Nie Y., Xie L., Song H.M., Gu Z.W. (2014). p53 mediated apoptosis by reduction sensitive shielding ternary complexes based on disulfide linked PEI ternary complexes. Biomaterials.

[26] Lee T., Lim E.K., Lee J., Kang B., Choi J., Park H.S., Suh J.S., Huh Y.M., Haam S. (2013). Efficient CD44-targeted magnetic resonance imaging (MRI) of breast cancer cells using hyaluronic acid (HA)-modified MnFe2O4 nanocrystals. Nanoscale Res. Lett..

[27] Miao W., Shim G., Kang C.M., Lee S., Choe Y.S., Choi H.G., Oh Y.K. (2013). Cholesteryl hyaluronic acid-coated, reduced graphene oxide nanosheets for anti-cancer drug delivery. Biomaterials.

[28] Kulig W., Jurkiewicz P., Olżyńska A., Tynkkynen J., Javanainen M., Manna M., Rog T., Hof M., Vattulainen I., Jungwirth P. (2015). Experimental determination and computational interpretation of biophysical properties of lipid bilayers enriched by cholesteryl hemisuccinate. Biochim. Biophys. Acta-Biomembr..

[29] Zhao C., Zhou B. (2022). Polyethyleneimine-based drug delivery systems for cancer theranostics. J. Funct. Biomater..

[30] Xu Y., Geng J., An P., Xu Y., Huang J., Lu W., Liu S., Yu J. (2015). Cathepsin B-sensitive cholesteryl hemisuccinate–gemcitabine prodrug nanoparticles: Enhanced cellular uptake and intracellular drug controlled release. RSC Adv..

[31] Cho S., Dang C., Wang X., Ragan R., Kwon Y. (2015). Mixing-sequence-dependent nucleic acid complexation and gene transfer efficiency by polyethylenimine. Biomater. Sci..

[32] Yang J., Huang L. (1998). Time-dependent maturation of cationic liposome–DNA complex for serum resistance. Gene Ther..

[33] Shi S.J., Han L., Gong T., Zhang Z.R., Sun X. (2013). Systemic Delivery of microRNA-34a for Cancer Stem Cell Therapy. Angew. Chem. Int. Ed..

[34] Li Y.Q., Guessous F., Zhang Y., DiPierro C., Kefas B., Johnson E., Marcinkiewicz L., Jiang J.M., Yang Y.Z., Schmittgen T.D. (2009). MicroRNA-34a Inhibits Glioblastoma Growth by Targeting Multiple Oncogenes. Cancer Res..

[35] Pang R.T.K., Leung C.O.N., Ye T.M., Liu W.M., Chiu P.C.N., Lam K.K.W., Lee K.F., Yeung W.S.B. (2010). MicroRNA-34a suppresses invasion through downregulation of Notch1 and Jagged1 in cervical carcinoma and choriocarcinoma cells. Carcinogenesis.

[36] de Antonellis P., Medaglia C., Cusanelli E., Andolfo I., Liguori L., De Vita G., Carotenuto M., Bello A., Formiggini F., Galeone A. (2011). MiR-34a Targeting of Notch Ligand Delta-Like 1 Impairs CD15(+)/CD133(+) Tumor-Propagating Cells and Supports Neural Differentiation in Medulloblastoma. PLoS ONE.

[37] Shi Q., Xue C., Zeng Y., Yuan X., Chu Q., Jiang S., Wang J., Zhang Y., Zhu D., Li L. (2024). Notch signaling pathway in cancer: From mechanistic insights to targeted therapies. Signal Transduct. Target. Ther..

[38] Nie F., Sun X., Sun J., Zhang J., Wang Y. (2025). Epithelial-mesenchymal transition in colorectal cancer metastasis and progression: Molecular mechanisms and therapeutic strategies. Cell Death Discov..

[39] Qian S., Wei Z., Yang W., Huang J., Yang Y., Wang J. (2022). The role of BCL-2 family proteins in regulating apoptosis and cancer therapy. Front. Oncol..

[40] Roy S., Zhu D., Parak W.J., Feliu N. (2020). Lysosomal proton buffering of poly (ethylenimine) measured in situ by fluorescent pH-sensor microcapsules. ACS Nano.

