# Supplementary material for: Cholesterol-Conjugated Polyion Complex Nanoparticles for Combination Delivery of Hydrophobic Paclitaxel and Hydrophilic miR-34a for Colon Cancer Therapy
Source: Int J Mol Sci. 2025 Aug 18;26(16):7965. doi: 10.3390/ijms26167965 (PMC12387032; doi:10.3390/ijms26167965)
Supplement: Supplementary file 1 [file ijms-26-07965-s001.zip › ijms-3749267-supplementary.pdf]

Supporting Information

Cholesterol-Conjugated Polyion Complex Nanoparticles for Combination Delivery of Hydrophobic Paclitaxel and Hydrophilic miR-34a for Colon Cancer Therapy

Arjaree Jobdeedamrong [1,2,†], Hye Jin Yoo [1,2,†], Hosun Jung [1,2,], Chiravoot Pechyen [3,4], Sitakan Natphopsuk [3,5], Peerapat Thongnuek [6], Seok Jeong [2,7], Junghan Lee [1,2,8,*] and Su-Geun Yang [1,2,8,*]

[1] BK21 FOUR Program in Biomedical Science and Engineering, Department of Biomedical Science,
Inha University College of Medicine, Incheon 22332, Republic of Korea; arjaree.j@inha.ac.kr (A.J.);
hyejin_yoo@inha.ac.kr (H.J.Y.); wjdghtjs1234@naver.com (H.J.)

[2] Translational Research Center, Biomedical Research Institute, Inha University Hospital, Inha University College of Medicine, Incheon 22332, Republic of Korea; inos@inha.ac.kr

[3] Thammasat University Center of Excellence in Modern Technology and Advanced Manufacturing for
Medical innovation, Thammasat University, Pathumthani 12120, Thailand; cpechyen@tu.ac.th (C.P.);
sitakan@tu.ac.th (S.N.)

[4] Department of Materials and Textile Technology, Faculty of Science and Technology, Thammasat
University, Pathumthani 12120, Thailand

[5] Chulabhorn International College of Medicine, Thammasat University, Pathumthani 12120, Thailand

[6] Biomedical Materials and Devices for Revolutionary Integrative Systems Engineering Research Unit (BMD-RISE), Faculty of Engineering, Chulalongkorn University, Bangkok 10330, Thailand; peerapat.t@chula.ac.th

[7] Division of Gastroenterology, Department of Internal Medicine, Inha University Hospital, Inha University College of Medicine, 27 Inhang-ro, Jung-gu, Incheon 22332, Republic of Korea

[8] Department of Biomedical Science, Inha University College of Medicine, 366 Seohae-daero, Jung-gu,
Incheon 22332, Republic of Korea

* Correspondence: jhlee02@inha.ac.kr (J.L.); sugeun.yang@inha.ac.kr (S.-G.Y.)

† These authors contributed equally to this work.

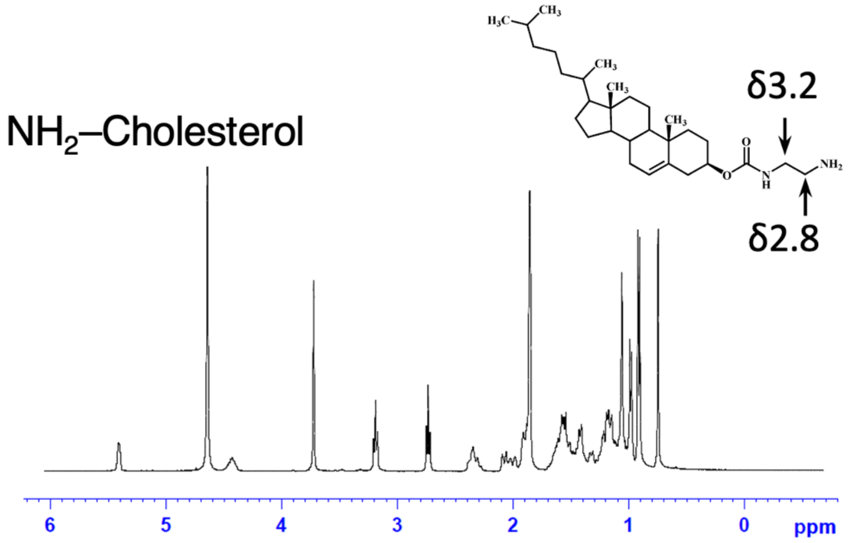

Figure S1. ^{1}H-NMR spectrum of amine–cholesterol.

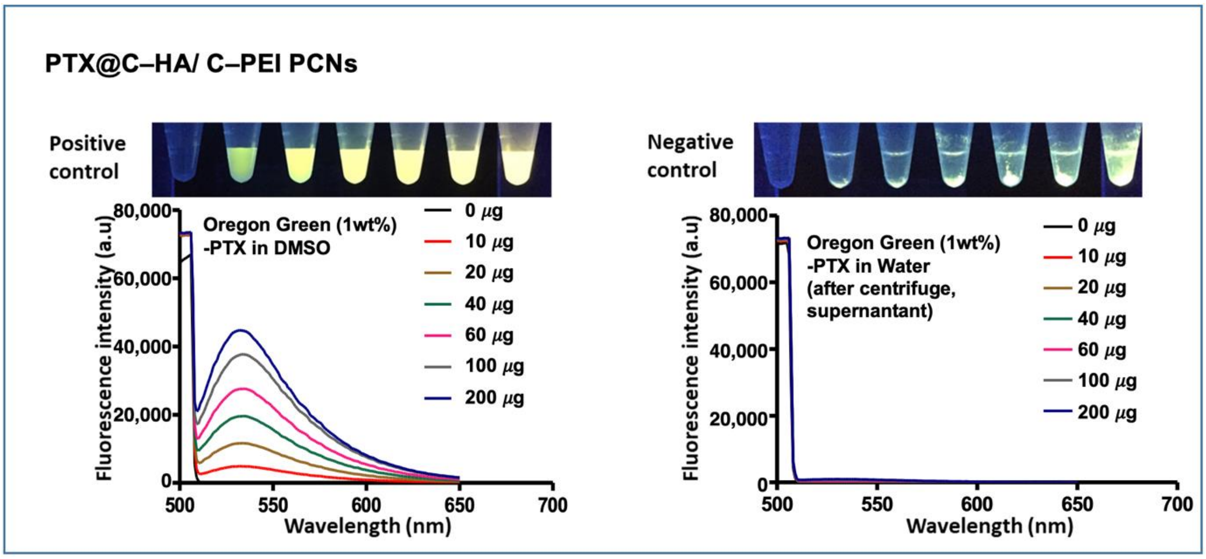

Figure S2. Fluorescence spectra and images of Oregon Green-labeled (1 wt%) paclitaxel (PTX) in DMSO (positive control) and in water containing cholesterol-conjugated polyion complex nanoparticles (C–HA/C–PEI PCNs) after centrifugation (negative control) at various PTX concentrations (0–200 μg).

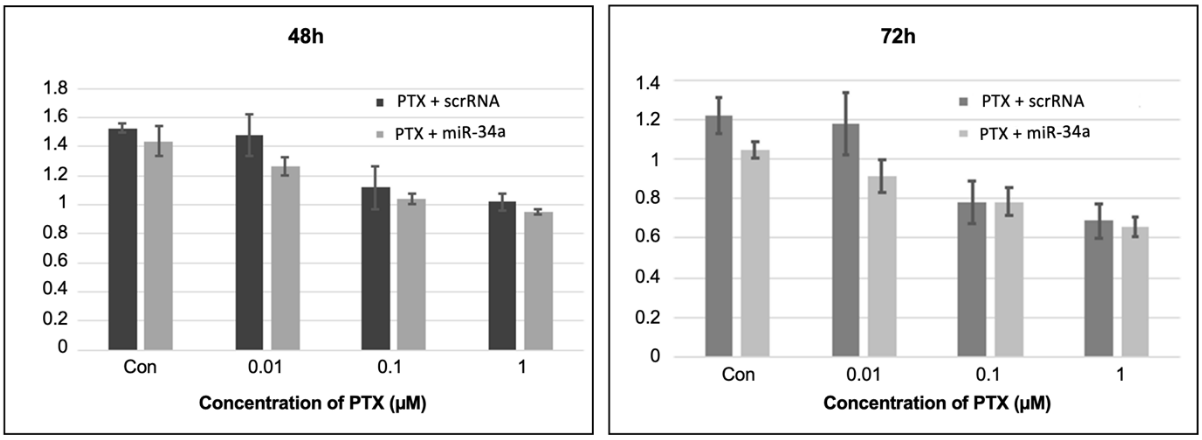

Figure S3. In vitro cell viability studies of dual-drug treatment.

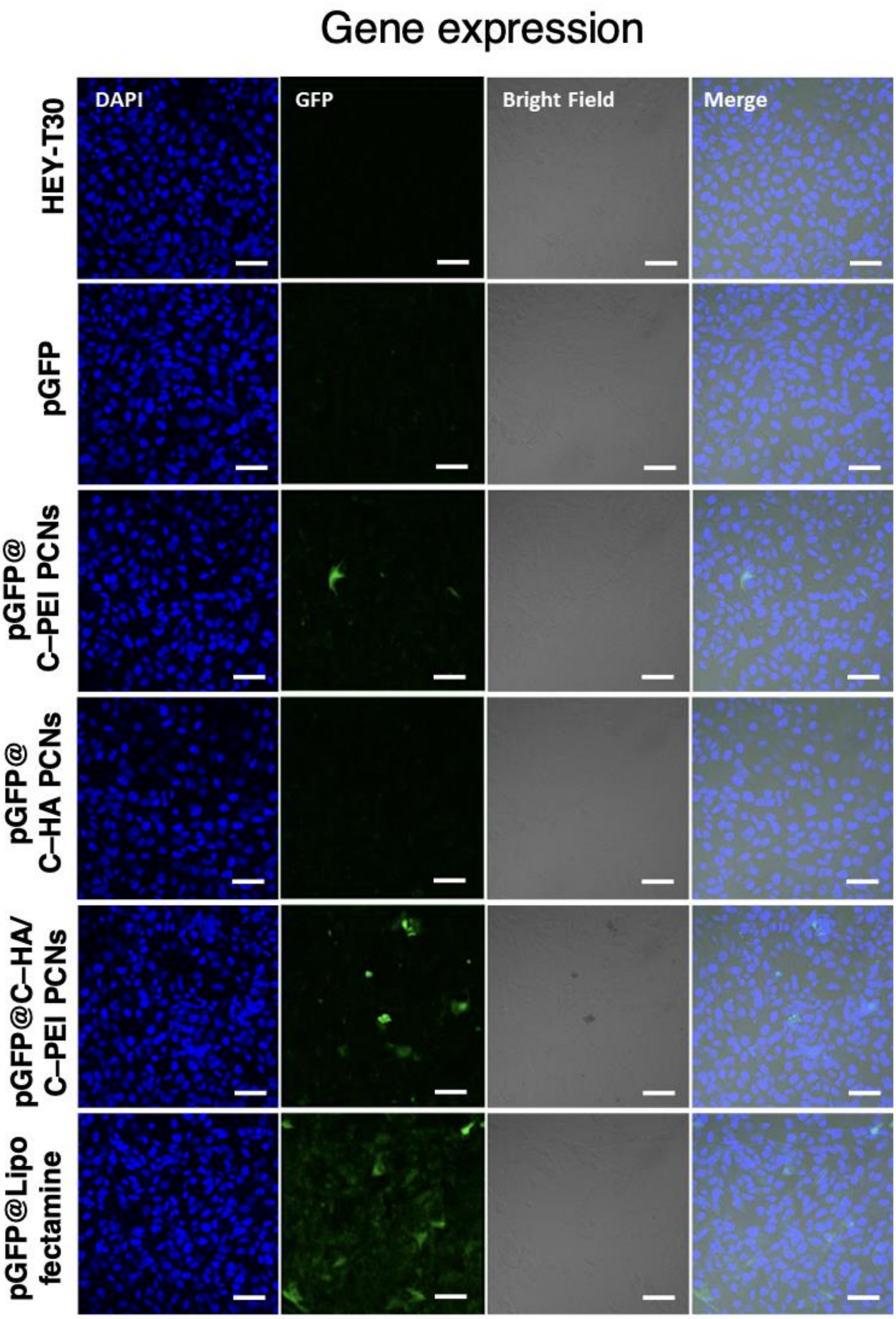

Figure S4. *In vitro* gene expression of PTX@C–HA/C–PEI PCNs. Scale bars, 50 μm.

Table S1 Hydrodynamic diameters and zeta potential of nanoparticles prepared with various concentrations of C–PEI and C–HA.

Entry	C–PEI (5ug/ml, ul)	pGFP (12.4 ug/ml, ul)	C–HA (5ug/ml, ul)	Hydrodynamic dimeter (nm)	Zeta potential (mV)
1	100	-	-	81 ± 4	15 ± 3.1
2	100	10	10	360 ± 8	6 ± 0.5
3	100	10	20	378 ± 141	6 ± 0.1
4	100	10	30	320 ± 123	-1 ± 0.3
5	-	10	10	150 ± 6	-24 ± 0.5
6	-	10	20	130 ± 12	-22 ± 1.5
7	-	10	30	81 ± 13	-21 ± 0.7
8	-	10	-		-12 ± 2.2
9	-	-	30		-11 ± 3.5
